# Anti-Müllerian Hormone and Polycystic Ovary Syndrome in Women and Its Male Equivalent

**DOI:** 10.3390/biomedicines10102506

**Published:** 2022-10-07

**Authors:** Nathalie di Clemente, Chrystèle Racine, Rodolfo A. Rey

**Affiliations:** 1 Centre de Recherche Saint-Antoine (CRSA), Sorbonne Université-INSERM, 75012 Paris, France; 2 Institut Hospitalo-Universitaire ICAN, 75013 Paris, France; 3 Sorbonne Paris Cité, Paris-Diderot Université, 75013 Paris, France; 4 Centro de Investigaciones Endocrinológicas “Dr. César Bergadá” (CEDIE), CONICET-FEI-División de Endocrinología, Hospital de Niños Ricardo Gutiérrez, Buenos Aires C1425EFD, Argentina

**Keywords:** anti-Müllerian hormone, Müllerian inhibiting substance, anti-Müllerian hormone, polycystic ovary syndrome, male polycystic ovary syndrome equivalent

## Abstract

This article reviews the main findings on anti-Müllerian hormone (AMH) and its involvement in the pathogenesis of polycystic ovary syndrome (PCOS) and its male equivalent. In women, AMH is produced by granulosa cells from the mid-fetal life to menopause and is a reliable indirect marker of ovarian reserve. AMH protects follicles from atresia, inhibits their differentiation in the ovary, and stimulates gonadotrophin-releasing hormone neurons pulsatility. AMH overexpression in women with PCOS likely contributes to the increase of the follicle cohort and of androgen levels, leading to follicular arrest and anovulation. In the male, AMH is synthesized at high levels by Sertoli cells from fetal life to puberty when serum AMH falls to levels similar to those observed in women. AMH is involved in the differentiation of the genital tract during fetal life and plays a role in Sertoli and Leydig cells differentiation and function. Serum AMH is used to assess Sertoli cell function in children with disorders of sex development and various conditions affecting the hypothalamic–pituitary–testicular axis. Although the reproductive function of male relative of women with PCOS has been poorly investigated, adolescents have elevated levels of AMH which could play a detrimental role on their fertility.

## 1. Polycystic Ovary Syndrome: Diagnostic Criteria, Main Traits, and Pathophysiology

Polycystic Ovary Syndrome (PCOS) is the most prevalent hormonal disorder affecting as many as 8–13% of women of reproductive age worldwide [1]. It is characterized by an irregular ovulatory function (oligomenorrhea or amenorrhea), the evidence of either biological and/or clinical hyperandrogenism, and the presence of a polycystic ovarian morphology [2]. The diagnosis of the syndrome requires the association of at least two of the aforementioned Rotterdam criteria. Four phenotypes have been described according to the combinatorial of these three features. Most patients with PCOS also exhibit high serum anti-Müllerian hormone (AMH) and Luteinizing Hormone (LH) levels [3]. Moreover, women with PCOS present at various degrees different metabolic disorders adding to the heterogeneity of this syndrome in its clinical presentation. Indeed, PCOS is generally associated with insulin resistance (IR), abdominal adiposity, high cholesterol levels, nonalcoholic fatty liver disease/nonalcoholic steatohepatitis, and high blood pressure, with an increased risk of type 2 diabetes mellitus (T2DM) and cardiovascular diseases [4]. Other multiple morbidities are linked with PCOS, including psychiatric disorders such as anxiety and depression [5], obstructive sleep apnea, and endometrial cancer [6]. Finally, women with PCOS have a high risk for developing gestational diabetes, preeclampsia, and preterm deliveries [7].

First degree male relatives of women with PCOS also present a male PCOS equivalent. The main traits observed in fathers, brothers, and sons of women with PCOS are metabolic disturbances such as impaired glucose intolerance, reduced insulin sensitivity, obesity, and dyslipidemia, resulting in a higher prevalence of T2DM and cardiovascular diseases [8,9,10]. As for women with PCOS, psychiatric disorders have also been reported [11]. An increased prevalence of early-onset (<35 years) androgenic alopecia (AGA) is observed in male relatives of women with PCOS, which has been proposed as a phenotypic sign of the male PCOS equivalent [12]. It is more likely that AGA is related to their high dehydroepiandrosterone, 17α-hydroxyprogesterone and low sex hormone-binding globulin levels, than to their testosterone concentrations for which the data are contradictory. Fewer studies have been performed on the reproductive function of these men. Only two articles analyzed the sperm parameters of adult sons of women with PCOS [13] and of patients with AGA [9], respectively, and did not find differences compared to controls. Higher levels of LH and AMH, and decreased concentrations of follicle stimulating hormone (FSH), have been also reported in male relatives of women with PCOS, although data are discordant [14,15,16].

Most of the reproductive and metabolic traits present in women with PCOS are transmitted to their children, since 60 to 70% of their daughters will manifest their own PCOS phenotype during adolescence and as young adulthood [17]. In the same way, daughters of women with PCOS have a 5-fold increased risk of developing PCOS themselves [18]. Several PCOS susceptibility genes or loci have been identified but these only account for a small percentage (<10%) of women with PCOS and cannot explain the heterogeneity of PCOS phenotypes, suggesting the involvement of other mechanisms. It is likely that epigenetic and environmental factors also contribute to the development of the PCOS [17,19,20]. In particular, cumulative evidence now indicates that the reproductive and metabolic disorders observed in women with PCOS might result from developmental alterations during fetal and pre-pubertal life, in accordance with the Developmental origins of Health and Disease concept. Among the factors involved in the development of the key features of PCOS, androgens and insulin/IR play a central role. In keeping with maternal androgenization of fetuses, an increased ano-genital distance [21] and a decreased expression of 3β-hydroxysteroid dehydrogenase (3β-HSD) in the placenta of women with PCOS have been reported. In line with these results and the heritability of the PCOS phenotype, Risal et al. [22] have shown a transgenerational effect of a fetal treatment with dihydrotestosterone (DHT), a nonaromatic androgen, in a PCOS-like mice model. In addition, a recent hypothesis has suggested that the high AMH levels in pregnant PCOS women could contribute to the androgenization of their fetuses, leading to PCOS in adulthood, and participate, through epigenetic modifications, in the transgenerational inheritance of this syndrome [23].

## 2. The Anti-Müllerian Hormone System

AMH is a 140 kDa homodimeric glycoprotein, belonging to the Transforming Growth Factor-β (TGF-β) family (reviewed in [24,25]). AMH signals through two distinct serine/threonine type I and type II receptors, with a single transmembrane domain. The ligand binding induces the phosphorylation of type I receptor kinase by the type II receptor. This leads to the phosphorylation of the cytoplasmic Small Mothers Against Decapentaplegic (SMAD) proteins, which interact with the common SMAD4 protein, and translocate into the nucleus where they control gene expression. AMHR2, the type II receptor, and AMH, are mutually specific, while AMH shares its type I receptors Activin receptor-Like Kinase (ALK)2/Activin A receptor type I, ALK3/Bone Morphogenetic Protein Receptor (BMPR)1A, and ALK6/BMPR1B, and SMAD proteins 1, 5, and 8, with the BMPs. AMH is synthesized as a large homodimeric precursor, which undergoes an obligatory proteolytic cleavage at monobasic sites, generating a non-covalent complex constituted by an N-terminal pro-region and a smaller C-terminal mature domain, that can bind to AMHR2 and initiate signaling. As is the case for the other members of the TGF-β family, depending on cell types, AMH can also activate other signaling pathways.

**Figure 1:**
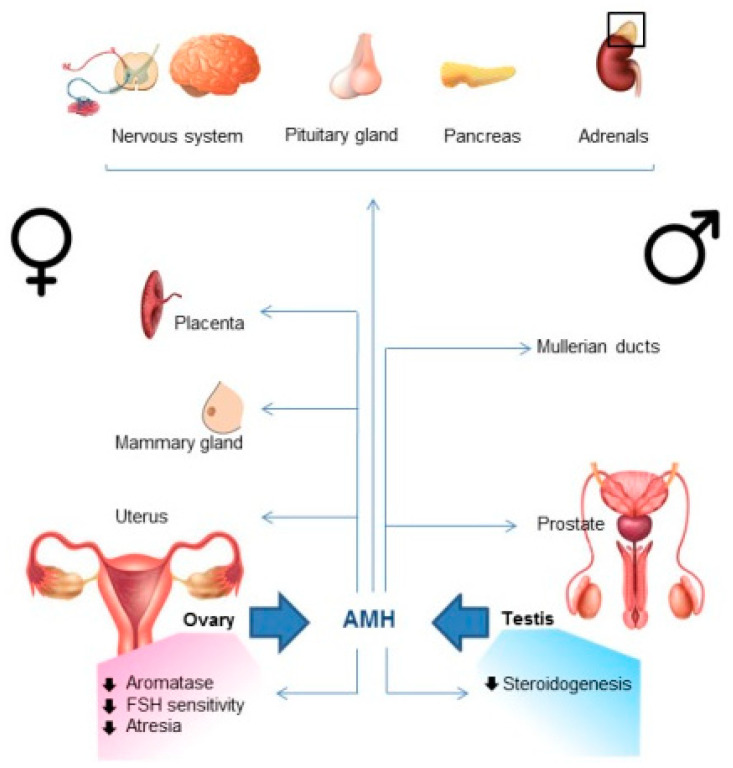
Main AMHR2-expressing organs and effects of AMH in the gonads. AMH is mainly produced by the gonads (large blue arrows). AMHR2-expressing organs (thin arrows) are the placenta, the mammary glands, the uterus, and the ovaries in females, and the Müllerian ducts, the prostate, and the testes in males. AMHR2 is also expressed in the nervous system, the pituitary gland, the adrenals, and the pancreas in both sexes. In the ovaries, AMH represses aromatase, follicle sensitivity to FSH and follicle atresia. In the testes, AMH inhibits steroidogenesis.

Circulating AMH is mostly of gonadal origin (Table 1, Figure 1). In women, AMH is expressed by the granulosa cells (GCs) of growing follicles, from the beginning of folliculogenesis to menopause (reviewed in [24]). In men, high levels of AMH are produced by Sertoli cells of the testes from fetal life to puberty, before decreasing to reach at adulthood concentration close to those observed in adult women (reviewed in [25]). Over the recent years, AMH has also been shown to be expressed by organs other than the gonads, such as the motoneurons [26], the neurons [27], the hypothalamus [28], and the gonadotrope cells of the pituitary gland [29]. However, this expression does not contribute to circulating levels since serum AMH reflects its expression profile in the gonads. The expression of AMHR2 is more widespread, supporting the relevance of the effects of AMH observed in AMHR2-expressing tissues (Table 1, Figure 1). Indeed, in females, AMHR2 has been mainly detected in GCs, but also in germ cells, theca cells and epithelial cells of the ovaries, and in the breasts, the uterus, and the placenta (reviewed in [24]). In males, AMHR2 is expressed by mesenchymal cell of the Müllerian ducts, Sertoli, Leydig (reviewed in [25]), and germ cells of the testes [30], but also in the prostate [31]. In addition, AMHR2 has been detected in the lungs [32], the motoneurons [26], the neurons [27], the hypothalamus [28], the gonadotrope cells of the pituitary gland [29], the pancreas [33], and the adrenals [33]. 

Serum AMH is now recognized as an important clinical marker for diagnosing and assessing reproductive disorders in both women and men. In women, serum AMH concentration is a predictive marker of ovarian reserve and ovarian response to controlled ovarian stimulation [36,37]. In boys, serum AMH can be used to evaluate Sertoli cell function in children with disorders of sex development [38] and various conditions affecting the hypothalamic-pituitary-testicular axis [39]. In the adult male, serum AMH usefulness is more limited than that of the other typical Sertoli cell marker, inhibin B. Nonetheless, it has been proposed to contribute to the management of azoospermia and the prediction of testicular sperm retrieval [40].

## 3. AMH and the PCOS

### 3.1. Overexpression of the AMH/AMHR2 System in Women with PCOS 

In ovaries of all species tested to date, AMH expression is initiated in primary follicles, is highest in pre-antral and small antral follicles, drops in large antral follicles, and becomes nearly undetectable except in cumulus cells (reviewed in [24]). AMH expression is not detected in luteal bodies and atretic follicles. Consistent with this expression profile, follicular fluid AMH concentrations are higher in small antral follicles than in larger ones. Serum AMH levels reflect the growing pool of follicles, and are low at birth, then increase in the post-natal activation period and reach maximal levels at the time of puberty, before gradually declining throughout reproductive life until menopause. The AMH-specific receptor AMHR2 is expressed as soon as fetal life, and then mainly co-expressed with AMH in the GCs of growing follicles.

Serum AMH levels are 2- to 4-fold higher in women with PCOS, as well as in daughters of women with PCOS. The elevated serum AMH levels in women with PCOS are due to both the enhanced number of small antral follicles, which express AMH the most, and an overexpression of AMH by their GCs [41]. AMHR2 is also up-regulated in GCs from women with PCOS compared to control one’s (reviewed in [24]). 

Modified receptivity of GCs to several hormones dysregulated in the PCOS could contribute to this overexpression of AMH and AMHR2. Indeed, androgens up-regulate in vitro *AMH* mRNA levels only in GCs from women with PCOS which overexpress the androgen receptor, and there is a large consensus that androgens and AMH levels correlate positively in patients with this condition but not in normo-ovulatory women (reviewed in [24]). Furthermore, LH has no effect in GCs collected from control women but stimulates AMH expression in GCs from patients with PCOS, and most research teams have shown that serum AMH is positively correlated with LH levels in women with PCOS. Conversely, estradiol (E2) reduces the AMH expression in GCs of control women but does not regulate this gene in GCs from women with PCOS. Since the regulation of the AMH expression by E2 has been shown to depend upon a certain estrogen receptors (ESR) ESR1/ESR2 expression ratio, the increase of this ratio in GCs from women with PCOS could prevent the inhibitory effect of E2 on the *AMH* mRNA levels observed in GCs from control women. Similarly, *AMHR2* mRNA levels are downregulated in GCs from control women by LH and E2 whereas they are not modulated by these hormones in GCs from anovulatory women with PCOS. 

Because AMH has been shown to increase gonadotrophin-releasing hormone (GnRH)-dependent LH pulsatility and secretion [28], its overexpression could further intensify this vicious cycle. Finally, when present, a dysregulation of LH and androgens synthesis in women with PCOS, could further exacerbate the phenomenon.

Metabolic and inflammatory-associated factors, which are also often dysregulated in women with PCOS might also contribute to the overexpression of AMH, although correlation studies have yielded conflicting results. Indeed, in vivo, an increase in non-covalent AMH complex, suggestive of increased AMH bioactivity, was associated with peptide-C in the serum of PCOS women [42]. Serum AMH was also positively correlated with the expression of the chemerin receptor, chemokine-like receptor 1, in GCs from women with PCOS [43]. In Goto-Kakizaki rats, a well-known model of T2DM, which also presents all the reproductive disorders of lean women with PCOS, AMH levels are elevated compared to control Wistar rats [44]. In addition, in vitro, Liu et al. [45] showed that insulin up-regulated *AMH* mRNA expression in human luteinized PCOS GCs in a dose-dependent manner. 

### 3.2. Role of the AMH/AMHR2 System in the Reproductive Defects of Women with PCOS

There is a general consensus that AMH plays a critical role in the selection of a dominant follicle and thus ovulation, by controlling the different steps of folliculogenesis, and by acting on various cell types of the hypothalamo–pituitary–ovary axis (Figure 1) (reviewed in [24]). 

Indeed, AMH has been shown to inhibit primordial follicle in rodents but this effect is still controversial in women (reviewed in [24]). In contrast, it is clear that AMH represses the differentiation of growing follicles during all folliculogenesis stages. In particular, AMH reduced the expression of aromatase (Cytochrome P450 Family 19 Subfamily A Member 1 (*Cyp19a1*) gene), the enzyme responsible for the conversion of androgens to estrogens, and LH receptor, two markers of GCs differentiation, both in vitro and in vivo [46]. Consistent with the inhibitory effect of AMH on follicle responsiveness to FSH, knock-down of AMH expression either in AMH null mice [47] or after passive AMH immunization in sheep [48], leads to an increased number of gonadotropin-dependent antral follicles. The down-regulation of the FSH receptor expression by AMH could be responsible for its inhibitory role on the sensitivity of GCs to FSH [49]. In addition, AMH could induce expression of miR-181b that targets adenylate cyclase 9, thereby decreasing cAMP levels, and resulting in a reduction in FSH sensitivity and/or a suppression of FSH signaling [46]. In gonadotropin-stimulated conditions, AMH mainly represses genes involved in GCs steroidogenesis, in particular *Cyp19a1*, whose regulation might be related to the stimulation by AMH of Forkhead box protein L2 [50]. It is worthy to note that in human pre-antral follicles, at follicular selection, a significant reduction in both AMH protein and mRNA expression correlate with an up-regulation of estradiol synthesis and mRNA expression of aromatase [51].

AMH also protects follicles from atresia by preventing GCs apoptosis. This effect was first based on indirect evidence, such as the absence of AMH expression in atretic follicles [52] and the fact that AMH null mice presented an increased number of oocyte remnants and atretic follicles [53]. Then, it was shown that AMH treatment reduced the number of atretic follicles both in vivo in pre-pubertal and adult mice [46,54], and ex vitro, in cryopreserved mouse ovaries, and in pre-pubertal human ovarian cortex transplanted in mice [55,56]. Conversely, knocking-down AMH expression in macaque pre-antral follicles negatively impacted follicle survival [57]. In addition, AMH was shown, at physiological concentrations, to have anti-apoptotic effects on cancer GC lines and primary cultures of mouse and human GCs [54,58], and integrative biology analyses of AMH target genes identified in the AT29C mouse GC line, highlighted 307 AMH target genes potentially involved in reducing GCs death [54].

In the ovary, AMH could also regulates the steroidogenesis of theca cells since AMH was shown to inhibit androgen production by ovine theca cells from small growing follicles stimulated by LH [48].

Moreover, AMH modulates ovulation at the hypothalamic level, since AMH can stimulate GnRH neurons pulsatility, and consequently contributes to the regulation of LH and FSH secretion by the gonadotrope cells of the pituitary gland. Indeed, AMH can activate, in a dose-dependent manner, the proportion of GnRH neurons necessary to trigger pituitary gonadotropin secretions [28]. Moreover, the injection of AMH in the lateral cerebral ventricle of the brain of female mice induces an increase in both LH pulsatility and secretion by gonadotrope cells, comparable to that required to produce an ovulatory surge.

**Figure 2:**
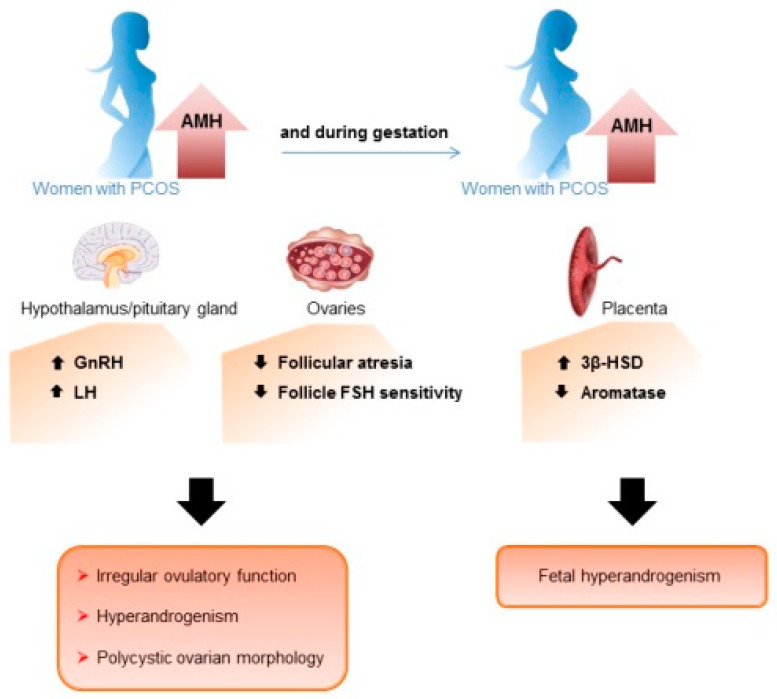
Involvement of AMH in the pathogenesis of the PCOS. In women with PCOS, the increased AMH levels (brown arrows beside the women with PCOS) may contribute to the development of their ovarian defects by acting both at the ovary and hypothalamo–pituitary complex. In pregnant women with PCOS, the increased AMH levels (brown arrows beside the women with PCOS) may in addition participate to fetal hyperandrogenism by disturbing placenta steroidogenesis. 🡇: inhibitory effect of AMH, 🡅: stimulatory effect of AMH.

In women with PCOS, the elevated levels of AMH and cleavage, the increased ability to bind AMHR2, and the overexpression of AMHR2 by PCOS GCs, strongly support the hypothesis that AMH effects are more pronounced (reviewed in [24]) (Figure 2). The facts that compared to ovulatory women with PCOS, anovulatory women with PCOS present AMH concentrations 18 times higher [59], with AMH levels correlated to the severity of the condition in terms of anovulation, and the fact that the AMH/AMHR2 system is dysregulated by LH only in GCs from anovulatory women with PCOS (reviewed in [24]), indicate that these increased AMH effects are related to menstrual disorders in patients with PCOS. Moreover, the fact that serum AMH levels are lower in PCOS women with a hyperandrogenic normo-ovulatory phenotype, compared to women with a non-hyperandrogenic oligo-anovulatory phenotype [60], shows that AMH can act independently of androgens on the ovulation defects of women with PCOS. 

In these patients, it is likely that increased AMH effects on follicle maturation enhance the inhibitory action of AMH on aromatase, and on the FSH-dependent effects involved in the process of follicles dominance and selection, leading to the follicular arrest (Figure 2). In addition, enhanced protective effects of AMH on follicular atresia may contribute to augment the number of follicles in the cohort as well as AMH levels, and then worsen the selection process of a dominant follicle. In keeping with the increased effects of AMH on follicle atresia in women with PCOS, several AMH target genes involved in reducing cell death, were over-expressed in GCs from women with PCOS compared to control cells [54]. In parallel, it is likely that the increased AMH action on, on one hand, follicle maturation and aromatase expression, and on the other hand, GnRH neurons pulsatility and LH production, contribute to enhance androgen levels in women with PCOS. The high levels of androgens in these patients would then stimulate primordial follicle growth and protect follicles from atresia, thus participating to increase the follicular cohort. Consistent with these effects of androgens, all the androgen-induced PCOS models display an increased number of growing follicles [61]. In women with PCOS, the inhibitory effects of AMH on primordial follicle recruitment and theca cells production of androgens (reviewed in [24]), if confirmed in human, could be counteracted by androgens and LH effects, respectively. 

In addition, increased AMH levels may affect other reproductive organs in women with PCOS. Consistent with this hypothesis, AMH was shown recently to modulate sexual behavior via an attenuated hypothalamic nitric oxide pathway, in an AMH-treated mouse model of PCOS [62]. Moreover, the expression of AMHR2 in the uterus [63] and the placenta [34] suggests that AMH might be involved in the pregnancy complications observed in women with PCOS, and which lead to abnormal gestational age and increased preterm deliveries [7].

### 3.3. Involvement of the AMH/AMHR2 System in the Origins of PCOS 

Since PCOS has a strong heritable component, with in particular, high AMH levels being also observed in the children of women with PCOS, *AMH* and *AMHR2* genes genetic studies have been carried out in several cohorts of patients with PCOS. However, the single nucleotide polymorphisms AMH Ile49Ser and AMHR2 -482A>G, which have been associated with PCOS [64], as well as the variants of *AMH* and *AMHR2* genes identified in 41 out of 608 women affected by PCOS [65], had reduced bioactivity. While these findings might explain increased theca cell testosterone production and primordial follicles recruitment in women with PCOS (reviewed in [24]), these effects could also be due to increased androgen and LH expression, respectively. In addition, a reduced action of the AMH/AMHR2 system is not in keeping with the other reproductive disorders present in women with PCOS. 

AMH was also suggested to play a part in the fetal programming of the PCOS, through an increased production of androgens by the placenta (Figure 2). Indeed, AMH treatment of mice at the end of gestation, stimulated GnRH and LH production, as well as androgens secretion, and led to the development of the main reproductive and neuroendocrine PCOS-like features in their female offspring [34]. In addition, the authors showed that if AMH cannot cross the placental barrier, AMH treatment decreased *Cyp19a1* expression in the placenta, which likely induces in utero hyperandrogenism. The same research team also showed epigenetic modifications of genes associated with PCOS in the third generation of these AMH-treated mice, consistent with the involvement of AMH overexpression in the in utero programming of PCOS [66].

## 4. AMH in Male PCOS Equivalent 

In the embryonic testes, AMH is detected in Sertoli cells as soon as these cells start to differentiate (reviewed in [25]). The expression of AMH is high during the rest of fetal life, and in boys until the beginning of puberty and the first signs of meiosis in seminiferous cords, and then decreases gradually, but is still detectable at adulthood. Comparison of seminal and serum AMH concentrations suggests that, after puberty, AMH is preferentially secreted by the apical pole of the Sertoli cell towards the seminiferous lumen [67].

The role of AMH has been mainly studied during male sexual differentiation, where AMH is responsible in male fetuses for the regression of Müllerian ducts, the anlagen of uterus, and fallopian tubes in females (Figure 1) (reviewed in [25]). Although testicular AMH expression stays high until puberty, the role of AMH after the Müllerian duct regression period in mammals remains unclear. The patients with the persistent Müllerian duct syndrome, due to mutations of either the *AMH* or the *AMHR2* genes, do not present fertility issues after removal of Müllerian derivatives, except in case of surgical complications [68]. This is also the case for patients with clinical conditions inducing high serum levels of AMH [38]. Similarly, the fertility of transgenic mice either lacking AMH or AMHR2, or overexpressing AMH, seems normal [69,70]. However, in vitro studies have shown that AMH down-regulates the expression of *Cyp11a1*, *Cyp19a1*, *Amhr2* [71,72], and stem cell factor [73] in immature Sertoli cells, and promotes their proliferation at low concentrations and apoptosis at high concentrations [73], suggesting that AMH may play a role in Sertoli cell development. In addition, AMH inhibits androgen production by both fetal and adult Leydig cells in cellular and mice models, acting by repressing steroidogenic enzymes (mainly P450 17α-hydroxylase/17,20-lyase), and by inhibiting Leydig cell differentiation [71,74]. 

Most studies reported increased AMH levels in either prepubertal sons or adult brothers and fathers of women with PCOS [13,16]. Adolescent male sheep exposed prenatally to androgen excess were also shown recently to present higher AMH levels than controls [75]. These elevated levels of AMH could be due to an increased number of Sertoli cells, as suggested by other androgenized animal models [76]. In addition, in line with an overexpression of AMH, reduced methylation of the *AMH* promoter has been detected only in sons (and not in girls) of women with PCOS [77]. As for women with PCOS, a dysregulation of AMH expression by hormones upregulated in male relatives of women with PCOS could also contribute to increase their AMH levels. The role of AMH in the reproductive function of males with PCOS equivalent has not been investigated up to now, but AMH could act on their Sertoli or Leydig cell functions. 

## 5. Conclusions

Numerous data have shown that AMH is involved in the ovulation defects observed in women with PCOS. AMH could also play a part in the origins of this syndrome. Several pieces of evidence indicate that AMH is overexpressed in the male PCOS equivalent, and whether AMH has effects on the reproductive function of male relatives of women with PCOS requires further investigations.

## Figures and Tables

**Table 1:** Distribution of AMH and AMHR2 in both sexes.

	Organs	Women	Men	Both	References
AMH expression	ovaries	++++			Reviewed in [24]
	testes		++++		Reviewed in [25]
	nervous tissue			+	[26,27]
	hypothalamus			+	[28]
	pituitary gland			+	[29]
AMHR2 expression	ovaries	++++			Reviewed in [24]
	testes		++++		Reviewed in [25]
	uterus	++			Reviewed in [24]
	placenta	+			[34]
	breasts	+			[35]
	prostate		+		[31]
	nervous tissue			+	[26,27]
	hypothalamus			+	[28]
	pituitary gland			+	[29]
	lungs			+	[32]
	pancreas			+	[33]
	adrenals			++	[33]

The number of + reflects the level of expression of AMH or AMHR2 in the different tissues.

## Data Availability

Not applicable.
